# Supplementary material for: The Bio-Persistence of Reversible Inflammatory, Histological Changes and Metabolic Profile Alterations in Rat Livers after Silver/Gold Nanorod Administration
Source: Nanomaterials (Basel). 2021 Oct 9;11(10):2656. doi: 10.3390/nano11102656 (PMC8538332; doi:10.3390/nano11102656)
Supplement: Supplementary file 1 [file nanomaterials-11-02656-s001.zip › nanomaterials-1384642-supplementary.pdf]

The bio-persistence of reversible inflammatory, histological changes and metabolic profile alterations in rat livers after silver/gold nanorod administration

Ying Liu [1], Hairuo Wen [2], Xiaochun Wu [3], Meiyu Wu [3], Lin Liu [3], Jiahui Wang [2], Guitao Huo [2], Jianjun Lyu [2,4], Liming Xie [3,5]* and Mo Dan [2,6]*

1 CAS Key Laboratory for Biomedical Effects of Nanomaterials and Nanosafety, NCNST-NIFDC Joint Labora-tory for Measurement and Evaluation of Nanomaterials in Medical Applications, Center for Excellence in Nanoscience, National Center for Nanoscience and Technology, No.11 Beiyitiao Zhongguancun, Haidian District, Beijing, 100190, P. R. China.; liuy1@nanoctr.cn

2 National Center for Safety Evaluation of Drugs, National Institutes for Food and Drug Control, No. 8 Hongda Mid-Road, Beijing Economic and Technological Development Zone, Daxing District, Beijing, 100176, P. R. China.; hairuowen@163.com (H.W.); jhwang18@mails.jlu.edu.cn (J.W.); lujianjun@nifdc.org.cn (J.L.); danmo543@163.com (M.D.)

3 CAS Key Laboratory of Standardization and Measurement for Nanotechnology, NCNST-NIFDC Joint Labora-tory for Measurement and Evaluation of Nanomaterials in Medical Applications, Center for Excellence in Nanoscience, National Center for Nanoscience and Technology, No.11 Beiyitiao Zhongguancun, Haidian District, Beijing, 100190, P. R. China.; wuxc@nanoctr.cn (X.W.); wumy@nanoctr.cn (M.W.); liulin@nanoctr.cn (L.L.); xielm@nanoctr.cn (L. X.)

4 Department of Pathology, InnoStar Bio-tech Nantong Co., Ltd., 226133 Nantong, P. R. China.; jjlv@innostar.cn

5 School of Nanoscience and Technology, University of Chinese Academy of Sciences, Beijing, 100049, P. R. China.; xielm@nanoctr.cn

6 The State Key Laboratory of New Pharmaceutical Preparations and Excipients, 226 Huanghe Road, Shijia-zhuang 050035, Hebei, P. R. China.; danmo543@163.com

* Correspondence: danmo543@163.com (M.D.); Tel.: 86-18600209506; xielm@nanoctr.cn (L. X.); Tel.: 86-10-82545722.

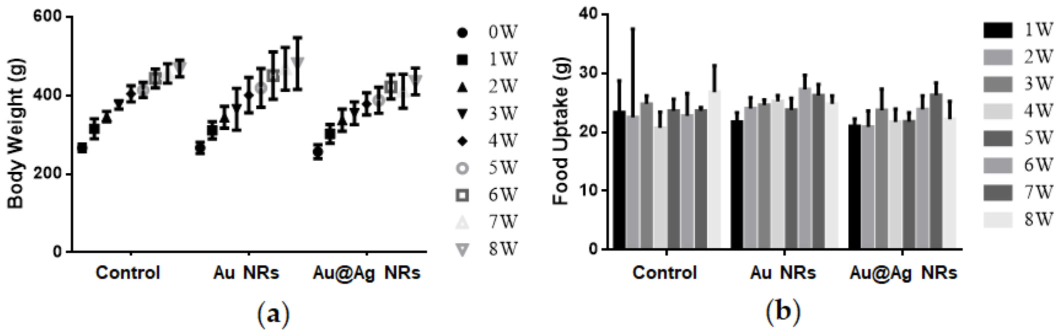

Figure S1 Effects of AuNRs and Au@Ag NRs on body weight and food ration. Body weight and food uptake of rats were monitored once per week for the 8W group after administration. And the average of body weight (a) as well as food uptake (b) was shown.

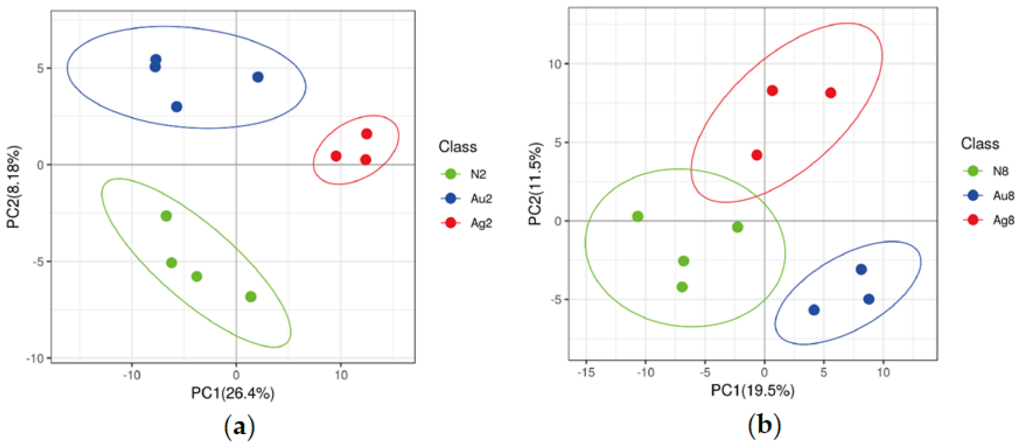

Figure S2 Score plots of PLS-DA revealing classifications. (a) 2 w. (b) 8 w.

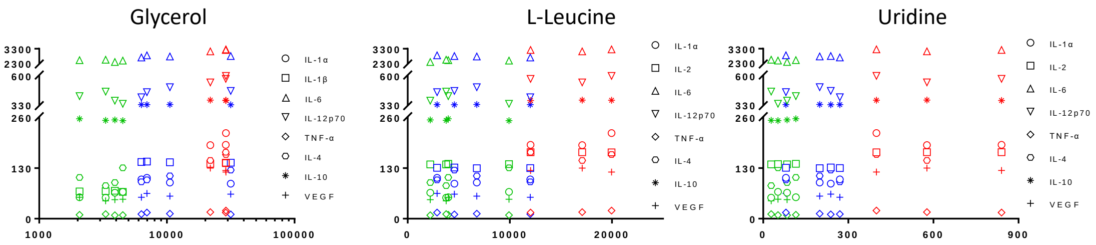

Figure S3 Correlations between metabolites and cytokines or growth factors at 2 w. Green, control mice. Blue, AuNRs-treated mice. Red, Au@Ag NRs-treated mice. Both X- axis and Y-axis were the peak area of metabolites.

Table S1 Hematological changes after the exposure of Au@Ag NRs. *$p < 0.05$ and $p < 0.01$ identified by Student's t-test, comparting to controls, respectively.

	1 w			2 w			4 w			8 w		
	Control	AuNRs	Au@Ag NRs	Control	AuNRs	Au@Ag NRs	Control	AuNRs	Au@Ag NRs	Control	AuNRs	Au@AgNRs
WBC (*10^9/L)	12.17±2.97	13.55±2.79	11.41±1.64	13.51±1.62	12.27±3.31	10.13±2.13	10.86±1.44	9.88±1.70	11.27±3.01	10.91±2.95	14.08±1.85	11.76±3.83
NEUT (%)	25.7±10.3	25.2±10.3	20.9±4.2	10.2±1.1	12.6±3.1	18.0±3.0*	19.6±9.6	16.2±3.8	21.1±12.6	11.0±4.7	11.3±2.7	12.55±7.42
NEUT (*10^9/L)	3.01±0.98	3.56±1.98	2.36±0.40	1.37±0.02	1.49±0.26	1.79±0.34	2.12±1.08	1.58±0.39	2.48±1.81	1.10±0.09	1.61±0.49	1.59±0.88
LYM (%)	69.8±9.9	66.7±10.4	73.9±4.7	84.3±2.6	80.5±3.9	76.4±4.3	72.7±12.8	78.6±4.5	74.7±12.7	84.0±4.8	82.9±3.1	80.88±6.35
LYMPH *10^9/L)	8.61±2.75	8.90±1.67	8.46±1.50	11.40±1.72	9.96±3.12	7.77±1.89	7.90±1.78	7.79±1.57	8.32±1.98	9.28±2.86	11.65±1.36	9.54±3.12
MONO (%)	2.3±0.4	2.5±0.7	2.7±0.5	3.8±1.3	3.8±1.0	3.2±0.9	2.3±0.4	2.5±0.4	2.0±0.4	2.7±0.6	2.7±0.6	2.63±0.56
MONO (*10^9/L)	0.29±0.08	0.49±0.19	0.31±0.08	0.50±0.12	0.44±0.04	0.32±0.07*	0.25±0.01	0.25±0.02	0.22±0.07	0.29±0.11	0.38±0.12	0.32±0.16
EOS (%)	0.5±0.4	2.8±2.2	0.8±0.3	0.5±0.1	0.7±0.2	0.6±0.2	4.2±3.9	1.2±0.6	1.2±0.7	0.7±0.2	0.9±0.3	0.95±0.79
EOS (*10^9/L)	0.06±0.03	0.36±0.24	0.09±0.04	0.06±0	0.08±0.03	0.06±0.03	0.48±0.45	0.12±0.04	0.14±0.08	0.08±0.04	0.12±0.04	0.10±0.05
BASO (%)	0.2±0.1	0.2±0.1	0.3±0.1	0.6±0.1	0.8±0.4	0.5±0.1	0.4±0.1	0.4±0.1	0.3±0.1	0.4±0.1	0.5±0.1	0.35±0.13
BASO (*10^9/L)	0.03±0.01	0.03±0.01	0.04±0.01	0.08±0.01	0.09±0.03	0.05±0.02	0.04±0.02	0.04±0.01	0.03±0.01	0.04±0.02	0.07±0.01	0.04±0.02
RBC (*10^9/L)	6.87±0.25	6.91±0.50	7.12±0.40	7.96±0.04	8.14±0.73	8.09±0.31	7.70±0.55	7.80±0.19	7.56±0.66	9.62±0.17	9.21±0.47	8.91±0.25*
HGB (g/L)	137±3	135±6	146±6	160±1	159±8	164±7	145±9	142±3	143±11	169±5 *	161±5	157±5*
HCT (%)	44.0±2.2	44.4±1.3	47.0±2.9	55.2±2.1	55.4±3.4	57.4±3.1	46.3±2.5	45.5±0.7	44.4±3.7	57.8±2.2	53.3±2.0	52.1±2.1
MCV (fL)	64.1±2.2	64.5±3.6	66.1±4.7	69.3±2.2	68.2±2.4	71.0±3.6	60.2±2.6	58.4±0.8	58.8±0.7	60.1±2.5	58.0±0.8	58.5±1.72
MCH (pg)	19.9±0.7	19.6±0.7	20.5±1.3	20.1±0.1	19.6±1.0	20.2±1.3	18.8±0.8	18.3±0.4	18.9±0.3	17.6±0.7	17.4±0.4	17.68±0.57
MCHC (g/L)	311±11	304±8	310±7	290±8	287±5	285±10	312±4	312±5	321±3*	292±3	301±3	302±6
PLT (*10^9/L)	1169±109	1006±237	1219±263	1005±43	980±123	1009±179	818±195	820±220	778±217	1123±125	1078±148	966±64
MPV (fL)	6.6±0.5	7.4±0.4	7.0±0.5	6.9±0.6	9.4±2.4	8.0±1.2	6.9±0.4	6.8±0.6	6.8±0.4	8.1±0.9	6.8±0.2	6.8±0.3
Retic (%)	5.84±0.69	8.65±0.72**	7.71±1.24*	3.31±1.15	3.41±0.92	3.77±0.75	2.99±0.72	3.08±0.89	3.36±0.56	2.74±0.47	2.54±0.36	3.36±0.44
Retic (*10^9/L)	401.1±50.0	594.6±29.2**	546.1±71.5**	262.8±90.4	275.8±70.4	306.1±67.0	227.0±36.6	240.1±66.9	257.4±35.4	264.1±46.9	233.8±36.8	298.1±31.8

Table S2 Effects of Au@Ag NRs on serum biochemical markers. *p < 0.05 and p < 0.01 identified by Student's t-test, comparting to controls, respectively.

	2 w			8 w		
	Control	AuNRs	Au@Ag NRs	Control	AuNRs	Au@Ag NRs
ALT (U/L)	51±6	43±8	48±6	50±9	45±8	40±5
AST (U/L)	98±12	84±4	86±2	95±11	83±2	85±4
ALP (U/L)	188±24	181±32	218±29	118±16	102±6	109±20
CK (U/L)	295±74	207±18	179±0**	196±19	163±22	221±168
LDH (U/L)	351±29	367±52	172±39*	380±41	318±53	304±265
TBIL (mmol/L)	1.08±0.10	1.06±0.20	0.85±0.38	1.18±0.44	0.97±0.71	1.50±0.14
UREA (mmol/L)	5.3±1.1	5.7±1.4	6.1±1.7	6.2±0.7	6.7±1.2	6.1±0.5
CRE (mmol/L)	27±3	25±5	25±3	33±7	34±3	32±6
GLU (mmol/L)	9.06±4.30	10.77±4.54	16.98±3.58	7.94±2.22	8.56±2.55	8.37±2.89
CHO (mmol/L)	1.86±0.43	1.50±0.35	1.78±0.26	1.92±0.24	1.63±0.21	1.68±0.47
TG (mmol/L)	0.40±0.08	0.49±0.11	0.77±0.16**	0.50±0.09	0.71±0.23	0.55±0.22
TP (g/L)	64.1±2.9	63.7±0.9	63.9±0.6	71.1±2.1	68.0±1.7	65.8±3.7*
ALB (g/L)	35.9±1.1	35.8±1.0	35.3±0.4	37.9±1.8	36.6±1.3	35.8±0.6
A/G (g/L)	1.28±0.10	1.29±0.08	1.24±0.03	1.14±0.08	1.17±0.09	1.21±0.11
K^+ (mmol/L)	8.75±2.69	9.38±1.94	9.98±0.93	11.44±2.66	7.74±2.21	8.55±2.18
Na^+ (mmol/L)	144.9±0.7	147.0±1.9	145.9±0.9	144.9±1.2	147.5±1.9	148.8±1.6**
Cl^- (mmol/L)	103.1±1.7	104.0±1.1	103.2±1.8	99.6±0.9	101.6±1.3	101.3±2.0
